# Biomechanical Characteristics of the Knee Joint during Gait in Obese versus Normal Subjects

**DOI:** 10.3390/ijerph19020989

**Published:** 2022-01-16

**Authors:** Fadi Al Khatib, Afif Gouissem, Raouf Mbarki, Malek Adouni

**Affiliations:** 1 Mechanical Engineering Department, Australian College of Kuwait, East Mishref, P.O. Box 1411, Safat 12000, Kuwait; fadi.alkhatib@ack.edu.kw (F.A.K.); afif.gouissem@ack.edu.kw (A.G.); raouf.mbarki@ack.edu.kw (R.M.); 2 Physical Medicine and Rehabilitation Department, Northwestern University, 345 East Superior Street, Chicago, IL 60611, USA

**Keywords:** knee osteoarthritis, OA, obesity, joint loading, gait, finite element method

## Abstract

Knee osteoarthritis (OA) is a growing source of pain and disability. Obesity is the most important avoidable risk factor underlying knee OA. The processes by which obesity impacts osteoarthritis are of tremendous interest to osteoarthritis researchers and physicians, where the joint mechanical load is one of the pathways generally thought to cause or intensify the disease process. In the current work, we developed a hybrid framework that simultaneously incorporates a detailed finite element model of the knee joint within a musculoskeletal model to compute lower extremity muscle forces and knee joint stresses in normal-weight (N) and obese (OB) subjects during the stance phase gait. This model accounts for the synergy between the active musculature and passive structures. In comparing OB subjects and normal ones, forces significantly increased in all muscle groups at most instances of stance. Mainly, much higher activation was computed with lateral hamstrings and medial gastrocnemius. Cartilage contact average pressure was mostly supported by the medial plateau and increased by 22%, with a larger portion of the load transmitted via menisci. This medial compartment experienced larger relative movement and cartilage stresses in the normal subjects and continued to do so with a higher level in the obese subjects. Finally, the developed bioengineering frame and the examined parameters during this investigation might be useful clinically in evaluating the initiation and propagation of knee OA.

## 1. Introduction

Osteoarthritis (OA) is a painful diarthrodial joint condition caused by insufficient and frequently abnormal healing of damaged joint tissue. OA is the most frequent kind of arthritis and rheumatism, and it is a major source of disability in seniors globally [1,2,3,4]. It is distinguished by slow and progressive articular cartilage degeneration, subchondral sclerosis, joint laxity, osteophyte production, and joint space constriction [5]. The knee is the most typically affected joint, with a far higher rate than the hip and ankle joints [6]. Despite the disease’s high prevalence, the sources of knee OA remain unknown. This is primarily because of the disease’s complex matrix, which includes an apparent modification in articular cartilage’s mechanical and metabolic properties, bone, and neuromuscular control [7]. In addition, mechanical variables, notably joint loading during daily activities, have been linked to the onset and progression of OA [8]. This is explained by the increased number of conservative interventions that improve certain loading-driven biomechanical parameters with very limited knowledge of disease progression [9,10,11]. Unhealthy weight is the greatest preventable risk factor for joint OA that has traditionally been connected to OA mechanical factors via increased compressive pressures in the knee joint during the stance phase. Moreover, obesity, on the other hand, has recently been linked to visible changes in joint kinematics and kinetics, particularly in the frontal and sagittal planes, which leads to increased abnormal loading during daily activities and subsequent knee joint degeneration [12,13,14,15,16,17,18].

According to the most recent published data (behavioral risk factor surveillance system: BRFSS), the global obesity rate is ten times higher than in 1975. Yet, surprisingly, the extent to which obesity impacts joint biomechanics remains unknown. Indeed, most earlier studies documenting lower extremity biomechanics in obese people have not offered a reliable quantitative assessment of several important variables of interest, such as muscle function and the distribution of relevant tissue stresses/strains among the joint [19,20,21,22,23,24,25,26,27]. This active-passive behavior helps explain the observed change in daily activity patterns [28]. The earlier investigations integrated in vivo measurement of joint kinematics with three-dimensional link-segment models to predict load within and around the joint and explicitly neglect the knee joint passive resistance.

We believe that effective prevention of knee OA requires a thorough understanding of stress distributions in various components under both intact and modified circumstances, such as obesity. Therefore, this study aims to develop a computational framework using finite element simulations and a musculoskeletal model of the lower extremity to understand the simultaneous interaction between changes in the basic properties of the human body (weight) and the aggregate mechanical behavior of the knee joint, particularly articular cartilage loading during the stance phase of gait. Achieving this goal will comprise a systematic engineering approach studying the obesity effect on joint biomechanics during simulated daily activities, which is critical for understanding short-term impairments as well as the potential long-term risk for joint degeneration. We hypothesized that muscle forces and knee tissue stresses changed clearly in the obese group (OB) compared with the normal-weight group (N) during the stance phase of gait.

## 2. Materials and Methods

### 2.1. FE Model

**Figure 1:**
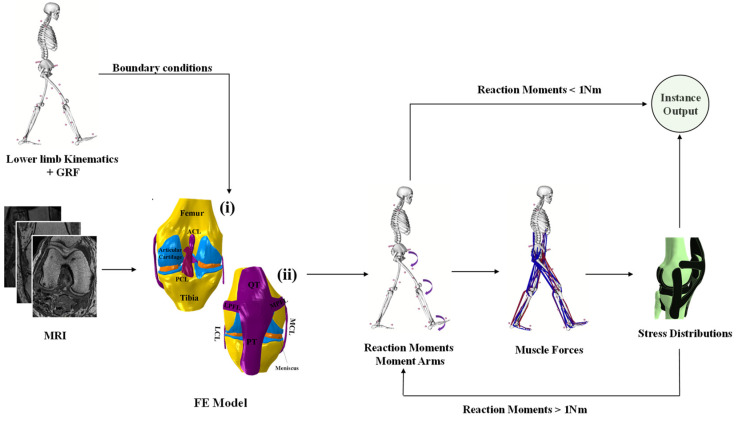
The workflow considered during this study with an anterior (i) and posterior (ii) view of the knee’s three-dimensional finite element model shows the corresponding soft tissues and articular surfaces acting on the bones. Anterior cruciate ligaments (ACLs), posterior cruciate ligaments (PCLs), medial and lateral collateral ligament (MCL and LCL), lateral patellofemoral (LPL), medial patellofemoral (MPL), quadriceps tendon (QT), and patellar tendon (PT) cartilage layers and menisci are shown. Muscles surrounding the knee joint were applied as surface traction [41,42]. More details on the system of axes, the joint center calculations, and muscles characteristics can be found in [29,40].

An anatomical model of knee joint including bones and their associated articular cartilage, meniscus, and ligaments (origins and insertions) was derived from a digitized magnetic resonance image (MRI) (Open Knee public domain repository at Simtk.org). The knee specimen (female subject: age = 70 years; height = 170 cm; weight = 77 kg) was scanned at Cleveland Clinic (Biomechanics laboratory) using one Tesla extremity MRI scanner (Orthone, ONI Medical Systems Inc, Wilmington MA). A scanning protocol that provided an optimal differentiation between the musculature, tendons, tissue fascia, and bone was used [29]. The image data set was then imported into 3D slicer 4.8 (MRI viewing and segmentation analysis package) and resampled in the anatomical planes. Using the HyperMesh (Altair Engineering, Troy, MI) pre-processor, polygonal elements were used to generate an FE mesh of the knee joint. The structure of the tibiofemoral joint was adjusted to match the given dimension in the open knee public domain repository at Simtk.org [29]. Reduced integration brick elements (C3D8R) were used to represent the articular cartilages, ligaments, and menisci. Bones were defined as rigid bodies using quadrilateral elements (S4R) that are in direct contact with elastic boundaries of the articular cartilages [30] (Figure 1). The model meshed with respect to 6% sensitivity analysis (difference in the mises stresses and principal strains) with an average side length of elements of 0.26 mm (312,719 elements). This developed knee model was integrated into a musculoskeletal model of the lower extremity, including the hip and ankle as spherical and hinge joints, respectively, as well as their surrounding muscles (Figure 1).

### 2.2. Constitutive Models of the Soft Tissue

#### 2.2.1. Cartilage

The articular cartilage was modeled using incompressible hyperelastic fibrils reinforced composites behavior described by Sajjadinia et al. [31]. The Cauchy stress ($\sigma^{c}$) in the model used was decomposed into non-fibrillar ($\sigma^{nf}$) and fibrillar ( $\sigma_{i}^{f}$) parts as follows:

(1) $$\left\{ \begin{array}{rlrr} \sigma^{c} & =v_{f}\sigma^{f}+\left(1-v_{t}\right)\sigma^{nf} & \\ \sigma^{nf} & =\eta_{0}^{s}\left[-\frac{\ln J}{6J}G_{m}\left(\frac{3\eta_{0}^{s}\ln J}{\eta_{0}^{s}-1}-3\frac{J+\eta_{0}^{s}}{J-\eta_{0}^{s}}-1\right)I+\frac{G_{m}}{J}\left(FF^{T}-J^{2/3}I\right)\right]+\frac{1}{D}(J-1{)}^{2}\\ \sigma_{i}^{f} & ={\left\{\frac{\eta_{0}^{s}}{J}\ln\varepsilon_{f}\left(E_{0}\varepsilon_{f}+E_{\varepsilon }\varepsilon_{f}^{2}\right)(n⨂n)\right\}}_{i} & \varepsilon_{f_{i}} & ≻0\\ \sigma_{i}^{f} & =0 & \varepsilon_{f_{i}} & ≼0 \end{array}\right.$$

where *F* and *J* are the deformation gradient tensor and the volumetric deformation, respectively. *n* and $\varepsilon_{f}$ are the current direction and logarithmic strain of the fibril, respectively. $E_{0}$ and $E_{\varepsilon }$ are the collagen stiffening coefficients (initial and strain-dependent). Gm is the shear modulus, $\eta_{0}^{s}$ is the initial solid volume fraction, and $v_{f}$ is the relative collagen fibril volume fraction. The collagen networks were defined as primary and secondary bundles of fibrils based on their orientation relative to the articular cartilage depth. The fibrils were oriented perpendicular to the subchondral junction and turned gradually in the middle zone to become parallel to the articular surface. For more details on the formulation of the material, please see prior works [31,32]. A list of the properties of the material is presented in Table 1.

#### 2.2.2. Meniscus

As the isotropy of the transverse and axial plans in the meniscus has been thoroughly described, a particular subclass of orthotropy, transverse isotropy, was employed to represent the mechanical behavior of this substance [33,34,35]. The local system axis of the meniscus is defined by axial, transverse, and circumferential axes, with the assumption that the transverse–axial plane is isotropic. As a result of this assumption, the number of independent constants in the matrix equals 5. To accomplish this, the transverse isotropy requires circumferential modulus (E_C_); transverse and axial modulus (E_t_ = E_a_); Poisson’s ratio (ν_ct_ = ν_ca_), which is defined as the ratio of the contractile strain in the transverse plane to the tensile strain in the circumferential direction under the load in the circumferential direction; and ν_ta_, which is the Poisson’s ratio within the transverse plane and shear modulus G. The stress–strain representation is defined as follows:

(2) $$\left\{\begin{array}{l} \varepsilon_{11}\\ \varepsilon_{22}\\ \varepsilon_{33}\\ \gamma_{12}\\ \gamma_{13}\\ \gamma_{23} \end{array}\right\}=\left[\begin{array}{cccccc} 1/Et & -vct/Et & -vta/Ec & 0 & 0 & 0\\ -vct/Et & 1/Et & -vta/Ec & 0 & 0 & 0\\ -vta/Et & -vta/Et & 1/Ec & 0 & 0 & 0\\ 0 & 0 & 0 & 1/Gt & 0 & 0\\ 0 & 0 & 0 & 0 & 1/Gc & 0\\ 0 & 0 & 0 & 0 & 0 & 1/Gc \end{array}\right]\left\{\begin{array}{l} \sigma_{11}\\ \sigma_{22}\\ \sigma_{33}\\ \sigma_{12}\\ \sigma_{13}\\ \sigma_{23} \end{array}\right\}$$

A list of the properties of the material is presented in Table 2.

#### 2.2.3. Ligaments

The knee tendons (PT and QT) were assumed to be neo-Hookean, with material coefficients (C10) of 55.9 MPa for the PT and 65.9 MPa for the QT [36]. Meanwhile, knee ligaments were modeled using an incompressible transversely isotropic hyperelastic behavior [37] via an uncoupled representation of the strain energy function [38]. The collagen fibers were uniformly distributed and properly bound to the isotropic and hyperelastic ground substance. The suggested strain energy function provides a silent reaction under any compressive loads and nonlinear stiffening behavior under tension as follows:

(3) $$\left\{ \begin{array}{l} \psi_{t}\left({\overset{-}{I}}_{1},{\overset{-}{I}}_{4},\overset{-}{J}\right)=\psi_{nf}\left({\overset{-}{I}}_{1}\right)+\psi_{f}\left({\overset{-}{I}}_{4}\right)\\ \psi_{nf}\left({\overset{-}{I}}_{1},\overset{-}{J}\right)=c_{1}\left({\overset{-}{I}}_{1}-3\right)+\frac{1}{D}(\overset{-}{J}-1{)}^{2}\\ \psi_{f}\left({\overset{-}{I}}_{4}\right)=\frac{c_{2}}{2c_{3}}\exp(c_{3}{\left({\overset{-}{I}}_{4}-1\right)}^{2})\;\;\;\mathrm{if}\;{\overset{-}{I}}_{4}>1\\ {\overset{-}{I}}_{4}={\overset{-}{F}}^{T}\overset{-}{F}:\left(n_{0}⨂n_{0}\right) \end{array}\right.$$

where $\psi_{nf}$, $\psi_{f}$, and $\;\psi_{vol}$ are the strain energy’s non-fibrilar, fibrillar, and volumetric parts, respectively. *n_0_* is the fiber orientation in the reference configuration; *F* is the deformation gradient tensor; *c_1_, c_2_,* and *c_3_* are the materials’ coefficients; and D is the incompressibility penalty parameter. The pre-strains’ behavior was incorporated into the ligaments by decomposing the deformation gradient (*F = F_0_ F_r_*) into a stress-free state (*F_0_*) and reference state (*Fr*). The pre-strains were defined here as the initial stretch ($\alpha_{0}$) field with *F*_0_ = $diago\left[\alpha_{0}\;\sqrt[{-1}]{\alpha_{0}}\;\sqrt[{-1}]{\alpha_{0}}\;\right]$ [39]. The current study considered the sets of material parameters from our recent publication [39].

### 2.3. Muscles Optimization

A nonlinear optimization technique was employed to evaluate the unknown muscle forces ({F}: vector of all lower limb muscle forces) at each instance of the stance phase of gait. This optimization procedure minimizes an objective function of the sum of cubed muscle stresses (f (Fi)) (4) under the constraint that muscle forces remain positive between their passive forces and total maximum active forces (6). The passive and maximum active force for all muscles were driven from a scaled musculoskeletal model that matched the female subject’s dimension considered to build the knee model [37]. The main constraint driving the evaluation of the muscles’ forces was the equations of equilibrium (5).

(4) $$f\left(F_{i}\right)=\overset{n}{\underset{i}{\sum }}{\left(\frac{F_{i}}{PCSA_{i}}\right)}^{3}$$

(5) [R]{F}={M}

(6) $$\left\{F_{p}\right\}\;\leq \left\{F\right\}\;\leq \left\{F_{max}\right\}$$

where $F_{i},PCSA_{i},\left[R\right],\left\{F_{p}\right\},\left\{F_{\;max}\right\}$ are the force and physiological cross-sectional areas of muscle i, lever arms matrix at different instances during stance phase, vector of passive muscle forces, and vector of maximum muscle forces, respectively [40]. {M} is the vector of the (required) lower limb joint moments (hip, knee, and ankle) computed during the different instances of the stance phase.

### 2.4. Loading and Boundary Conditions

**Figure 2:**
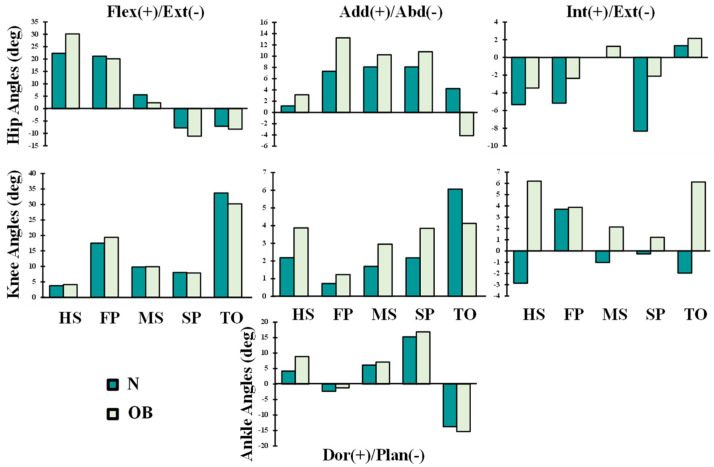
Lower extremity joints’ angles reported as the mean of normal-weight (N) and obese (OB) subjects during the stance phase of gait [43]. Five instances corresponding to the beginning heel strike (HS), first loading peak (FP), midstance (MS), second loading peak (SP), and toe-off (TO) of the stance phase are indicated.

**Figure 3:**
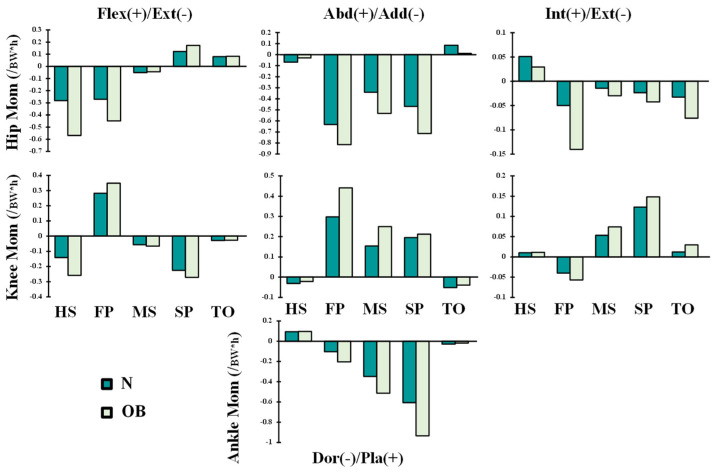
Lower extremity joints’ moments reported as the mean of normal-weight (N) and obese (OB) subjects during the stance phase of gait [43]. Loads were normalized to the body weight (BW = 732.365 N) and height (h = 170 cm) of the female subject of our FE model.

**Figure 4:**
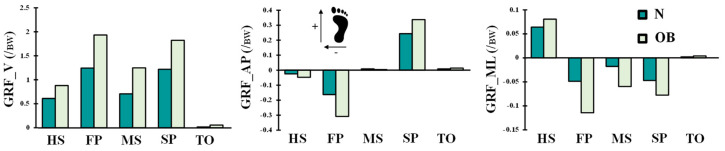
Vertical, anterior, and lateral ground reaction force (GRF) components at different stance phase times for normal-weight (N) and obese subjects (OB). Loads were normalized to the BW = 732.365 N of the female subject of our FE model.

The hip/knee/ankle joints kinematics (Figure 2) and kinetics (Figure 3 and Figure 4) during gait were based on reported in vivo measurements for asymptomatic (normal, N) and obese (OB) subjects [13,43]. Analyses were performed at five instances of the stance phase of gait (HS; heel strike, FP: first loading peak, MS: midstance, SP: second loading peak, and TO: toe-off). The femur was fully constrained at its instantaneous position for each instance, while the tibia was under prescribed rotations and the patella was entirely free. Next, the ground reaction force and leg/foot weights were applied to regenerate the joint reaction moments [43]. After that, unknown forces in muscles were iteratively approximated and applied as additional external loads to the model by updating the optimization algorithm with residual reaction moments and edited muscle lever arms owing to the passive resistance (Figure 1) [39,44]. The convergence is obtained when the needed moments fall below 1 N.m. Abaqus statics analysis and Matlab genetic algorithm were used.

## 3. Results

**Figure 5:**
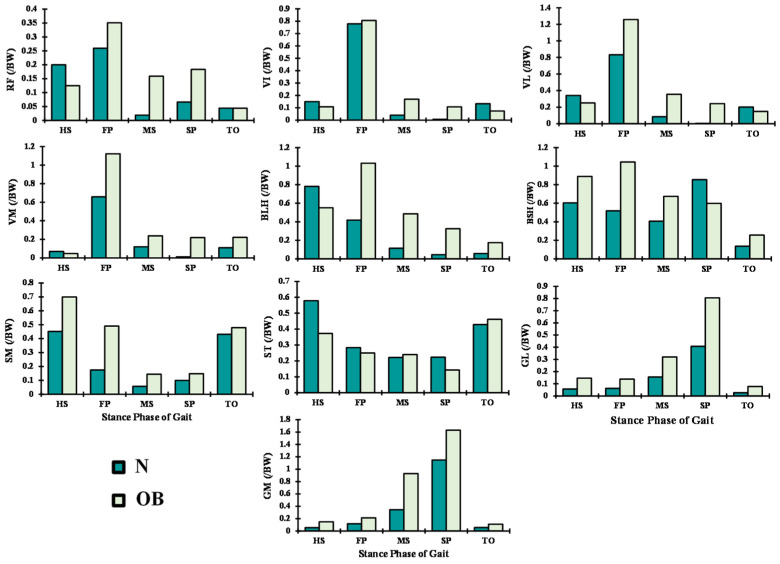
Predicted muscle forces at different periods of stance phase time for normal (N) and obese subjects (OB). Quadriceps: rectus femoris (RF), vastus intermidus (VI), vastus medialis (VM), and vastus lateralis (VL); hamstrings: biceps femoris long and short head (BLH and BSH), semimembranous (SM), and semitendinosus (ST); gastrocnemius: medial (MG) and lateral (LG).

In the OB case and as compared with the asymptomatic case, muscle forces in the quadriceps increased significantly by 63% (average) throughout the stance phase of gait (Figure 5). The activity of this muscle was more affected at the FP instance, where the vastus lateralis and medilas components were augmented by 34% and 87%, respectively. Furthermore, the lateral hamstring (BLH and BSH) was mainly altered at the FP and MS instances with argumentation that exceeded 62%. The maximum activity of this muscle was shifted with the obese subjects from the HS to FP instance (Figure 5). Except at the HS and FP periods, medial hamstrings (SM and ST) slightly altered with the obese cases. Forces in gastrocnemius fascicles increased by 37% at the MS period and 58% at the SP period.

**Figure 6:**
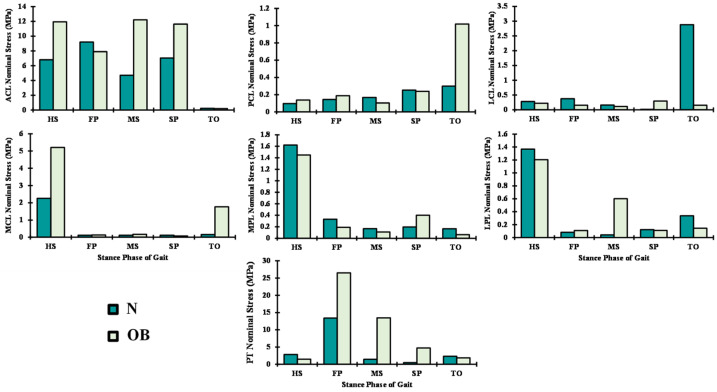
Predicted nominal ligament stresses at different periods of stance phase time for normal (N) and obese subjects (OB).

Among the tendons and ligaments, the patellar tendon loading follows the same trend of the quadriceps forces by reaching its maximum of nominal stress at FP in both groups (N and OB) with a clear increase of 52% with the OB group (Figure 6). The ACL nominal stresses throughout the stance phase of gait were the highest compared with the rest of the ligaments. LCL, PCL, and the patellofemoral ligaments (LPL and MPL) were loaded in the TO instance, while in contrast, the MCL was loaded at the HS (Figure 6). In most simulated instances, except the LCL and patellofemoral ligaments (LPL and MPL), the weight factors significantly increased the load on the ligaments; as an extreme example, the load on the ACL exceeded double at MS with the OB group (Figure 6). 

**Figure 7:**
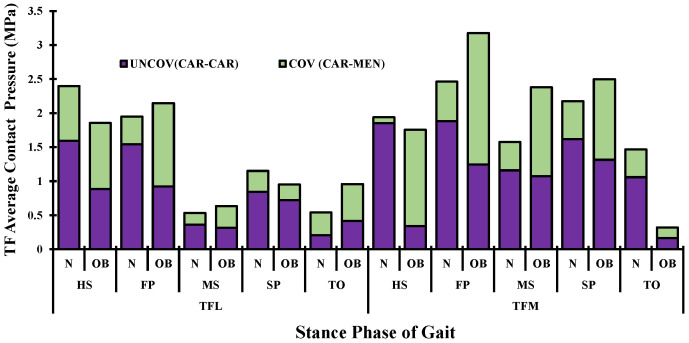
Predicted tibiofemoral (TF) average contact pressure at different periods of stance phase time for normal (N) and obese subjects (OB) (UNCOV: via cartilage, COV: via menisci, L: lateral, M: medial plateau).

**Figure 8:**
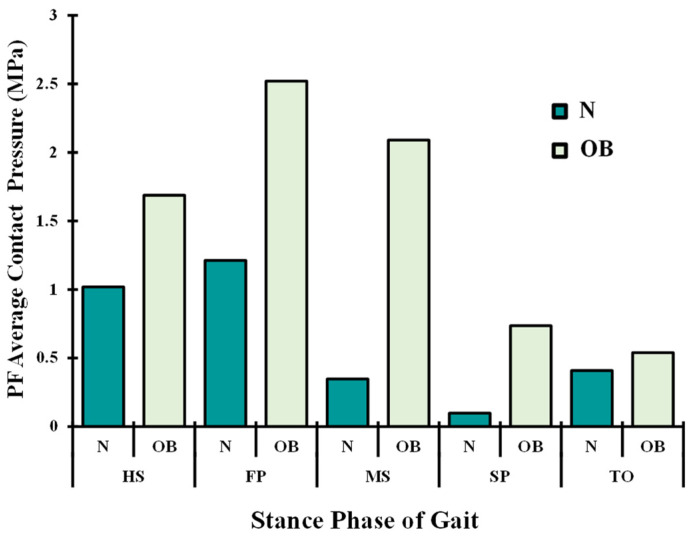
Predicted patellofemoral (PF) average contact pressure at different periods of stance phase time for normal (N) and obese subjects (OB).

**Figure 9:**
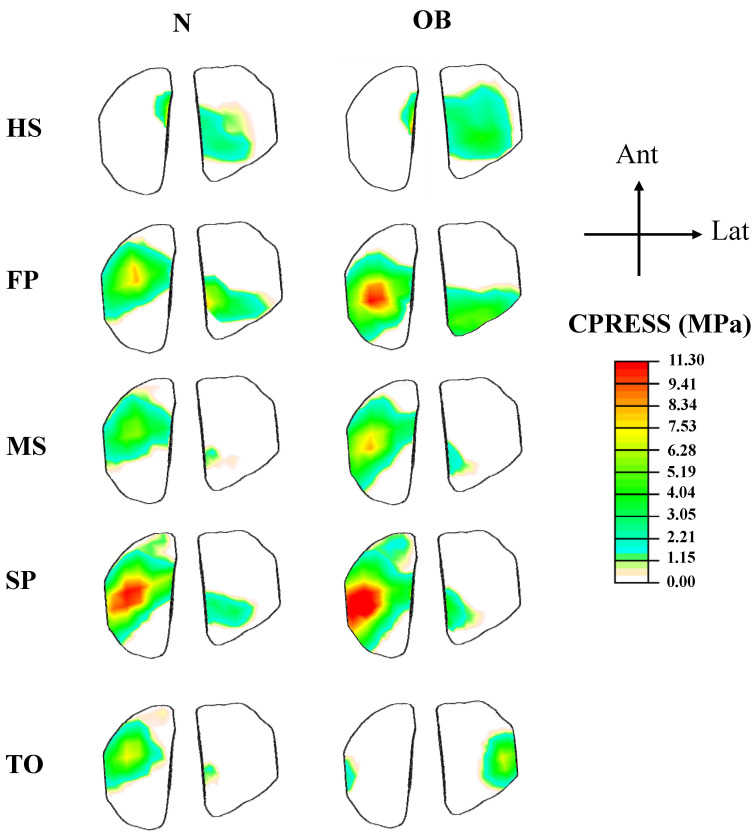
Contact compressive stress at articular surfaces of tibial compartments at different instances in normal and OB models. The common legend is employed for easier comparisons.

Following the variations in muscle activations, a large total tibiofemoral contact load was computed and transferred through tibial plateaus via covered (cartilage–cartilage) and uncovered areas (cartilage–meniscus) of articulation (Figure 7). This tibial load reached its maximum at FP and SP periods with a non-uniform distribution between the two plateaus (medial and lateral), where a much larger load was observed in the medial plateau at FP instance and thereafter. Compared with the normal case N, contact average pressures in OB were higher except at the start and end stance periods (HS and TO). A maximum difference of 39% between the two groups was computed on the medial side at the MS instance. Moreover, the proportion of average stresses transmitted via menisci was augmented in the OB case. The PF contact average stresses increased substantially during all of the periods owing to alteration in quadriceps forces in the OB group (Figure 8). The tibiofemoral and patellofemoral areas of contact showed similar trends as their respective contact load. Contact pressures distribution was shifted from the lateral plateau to the medial one after the HS, except at TO in the OB group. This distribution moved posteromedially from the FP to SP periods. The weight factor (OB) increased the peak articular contact pressure throughout the stance phase of gait and reached its maximum of 11.3 MPa at the SP period (Figure 9).

## 4. Discussion

This study investigated the alteration in the knee mechanical environment associated with the obesity factor during the stance phase of gait. For this purpose, a lower-extremity toolbox connecting a musculoskeletal model to an active-passive knee FE model was developed. This model was driven by separate kinematics-kinetics data of gait collected on normal weight and obese subjects [13,43]. To the best of our knowledge, this is the first study investigating the effect of obesity on the passive-active response of the knee joint in gait. Predictions confirmed the hypothesis that muscle forces and knee tissue stresses changed clearly in the OB group as compared with the normal group during the stance phase of gait.

In accordance with the clear augmentation of the knee extension moment and rotation at the early stance instance (HS) (Figure 2 and Figure 3), quadriceps muscle forces decreased at this period by an average of 21% (Figure 5) in the obese group. The most affected component of the quadriceps was the rectus-femoris muscle, with a percentage of reduction in its activity of 33%. This could be explained by the quadriceps being more efficient in generating flexion rather than extension moments present in the OB case at this gait period. Meanwhile, the trends in knee sagittal moments reversed to flexion with the rest of the simulated instances, resulting in significantly greater activation of quadriceps muscles in both subjects (N and OB) (Figure 5). The load in these muscles increased significantly with the OB group owing to the remarkable augmentation of the knee flexion moment and the unchanged kinematics of the knee joint in the sagittal plane. The maximum of this alteration was observed with vastus components, where vastus medialis force increased from 0.69 BW in the normal weight subject to 1.12 BW in the obese subject at the FP instance. The larger knee adduction moment at this instance may help in explaining the substantial augmentation of the vastus medialis activity [45]. At TO and despite no observed difference in flexion moment between the two groups, smaller quadriceps forces were computed in the OB group, likely due to the lower flexion angle [46].

In association with adduction and extension knee moment, predicted forces in the lateral hamstring (BLH and BSH) significantly decreased during the stance phase except at early periods (HS and FP) in all studied cases. These muscle activations increased in the OB subjects by 0.2 BW and 1.1 BW at HS and FP periods, respectively, compared with the normal-weight subjects (N). This higher activation level continues with the rest of the simulated periods to counterbalance the augmented adduction moment in the knee joint, which is often associated with OB subjects [47]. On the internal side of the knee joint, the muscle forces in the medial hamstring (SM and ST) decreased significantly except at the TO period as a response to the computed drop in the activation of the lateral hamstring. Besides, the augmentation of the hip extension and adduction moments with OB subjects represents an additional factor that conduced to the predicted augmentation in the hamstring muscle forces at early stance. The dominant lateral pattern of the hamstring muscles activation during the stance phase may aid in controlling lateral knee joint opening and provide greater knee stability against the prevailing adduction moments [48]. Despite the considerable adduction moments on the knee joint in both normal-weight and OB subjects, higher activity was computed in the MG as compared with the LG (Figure 5). To balance this frontal antagonistic activity, additional forces were estimated in lateral hamstrings mainly carried by the short-head component of the biceps femoris. The larger activity of the MG and the gastrocnemius, in general, was also linked to the higher dorsiflexion moment in the ankle joint at late stance (Figure 3).

In comparison with normal-weight subjects, the OB subjects were generally characterized by greater muscle activation and co-contraction levels via superficial EMG measurements [19,49,50]. The trend of computed muscle forces was compared with reported normalized EMG measurements [19,49,50]. Overall, the predictions in absolute terms in the normal-weight and OB groups with their associated variations matched the measured trends. However, estimated muscle forces in both the normal and OB groups were consistently lower than measurements at certain instances owing to the lack of consideration of coactivity in our FE model. This coactivity may help enhance joint stability and control during the stance phase of gait [51]. Moreover, it should be noted that the superficial collected EMG data and their normalization to the isometric maximal voluntary exertion should be taken with ultimate caution in the case of an obese subject. This later was characterized by a high mass of fat surrounding the regular mass of muscle, which may significantly affect the measured activation, and hence complicated any tentative validation of accurate muscles’ load [52]. Finally, the expected errors in superficial EMG measurements in deeper and larger muscles, as well as any attempt of mapping between normalized EMG magnitude and muscle load, are additional factors that warrant caution in such comparisons.

Owing to the changes in the muscle forces between normal-weight (N) and obese subjects (OB), predicted ligament nominal stresses altered relatively, especially in ACL. This stress increased by 48% in the OB subjects during the stance phase of gait, except at FP and TO periods. The remarkable higher activity in lateral hamstring muscles at FP and TO instances and the associated increase of the knee flexion angle may explain the observed drop in the ACL nominal stresses. As expected, the PCL stressed slightly at the TO period in the OB case owing to the additional joint internal rotation and activity of the medial hamstring. However, in response to the reduction of abduction moments and angles at the TO, the LCL stress dropped in the OB subjects. Low nominal stresses were computed in both groups (N and OB) in the patellofemoral ligaments (LPL and MPL), which may be linked to the relatively small flexion angle and the slight difference in the load distribution between the vastus components during the stance phase of gait. A similar variation of ligaments loading has been reported in earlier modeling studies [28,53,54,55,56].

The current investigation estimated average contact pressure in both the tibiofemoral and patellofemoral joints. Overall, the results of this analysis showed a substantial increase in the tibiofemoral loading in the OB subjects when compared with normal-weight subjects. The relative peak of average contact pressure occurred at the FP instance and was substantially augmented with OB subjects (~26%) (Figure 7), and this is due to the substantial increase in the surrounding muscles’ load and the associated higher axial joint forces. This result is in satisfactory agreement with earlier observations in the literature [20,23,57]. The medial compartment supports most of the load transmitted through the knee joint during the stance phase of gait with a slight increase in the supported load by the lateral compartments in the OB case at late stance (TO). The portion of the load transmitted via menisci increased in the OB group. In keeping with alteration of the contact loading, the medial plateau experienced larger compressive stress as well as a posteromedial shift in the center of the contact in all simulated cases during the stance phase of gait (Figure 9). A higher peak and a larger area of distribution of contact stresses were computed with OB subjects. Moreover, this distribution was shifted more in the posteromedial direction owing to the increased adduction and internal joint angles. The predicted locations of high cartilage stress with OB subjects are in agreement with observed MRI cartilage degeneration areas [58,59]. This observation may help explain the greater association between the BMI factor and the higher risk of developing knee OA [60]. Finally, in the comparison between the OB and normal-weight subjects, a clear augmentation in the cartilage stress in the patellofemoral joint was predicted with the OB case throughout the stance phase of gait. However, the stress on this joint is relatively small compared with the tibiofemoral one, which may explain the less concern given to the patellofemoral interaction in the literature [61]. 

In addition to the reported maximum stress concentration in cartilage and its relative, concluded role in risk evaluation of the knee OA initiation and progression, the musculoskeletal FE analysis proposed in this investigation may help understand the earlier observed OA reduced risk associated with weight loss [62] if it is simulated under the proper boundary conditions. Furthermore, scaling this simulation pipeline via the measured subject’s anatomical dimensions and driving the mechanical knee reaction by the same subject 3D motion capture, as well as considering tissue regular and degenerated responses, will provide the clinicians and therapists with an innovative tool that may help design the subject’s rehabilitation protocol. Furthermore, this type of conservative management based on tissue mechanics within different joint regions could be generalized to a wider population of knee management disorders where the mechanical factor can play a role [63]. 

The results and discussion in the current work should be considered in light of some limitations. Both normal-weight and obese subjects were assumed to have the same musculature without considering the fat mass. This later was negatively correlated with muscles’ activation in a very limited number of investigations in the literature [64]. However, if clear and agreed data were available, this factor could be considered with the proposed model. Muscle coactivity was not considered. The same lower-limb geometry was employed in this study for both groups. The measured kinematics-kinetics used as input data into our normal-weight and OB models may strongly influence the current results and conclusions. Despite the non-clear consensus on the differences in lower extremity kinematics between the healthy weight and obese subjects, the results of Russell [43] were used here owing to the large number of subjects in each group and the completeness of reported kinematics-kinetics data of the lower-extremity.

## 5. Conclusions

In conclusion, obesity-related changes in joint kinematics-kinetics at lower extremity measured during the stance phase of gait influenced lower extremity musculature activation as well as ligaments’ loading. In addition, articular cartilage contact stresses increased significantly in mean and peak components with obese subjects. Thus, the developed bioengineering frame and the examined parameters during this investigation might be useful clinically in evaluating the initiation and propagation of knee OA.

## Figures and Tables

**Table 1:** Article cartilage materials’ properties.

Material Parameters	
$E_{0}\left(MPa\right)$: Initial collagen coefficients	4.63
$E_{\varepsilon }\left(MPa\right)$: Strain-dep collagen coefficients	3670
$G_{m}\left(MPa\right)$: Shear modulus	0.723
$v_{f}$: Collagen fibril volume fraction ^1^	$v_{t}\frac{3}{13}$ or $v_{t}\frac{1}{13}$
$v_{t}$: Total depth-dependent collagen volume fraction ^2^	$1.4z^{2}-1.1z+0.59$
$\eta_{0}^{s}$: Initial solid volume fraction	0.1z+0.1
D: Incompressibility penalty parameter	0.0001

^1^$\frac{3}{13}$ for the primary fibril and $\frac{1}{13}$ for the secondary one. ^2^ z: Normalized depth of the articular cartilage (starting from the cartilage–bone junction area).

**Table 2:** Meniscus materials’ properties.

Ec (MPa)	Et (MPa)	$\nu_{ct}$	$\nu_{ta}$	Gt (MPa)
120	20	0.3	0.2	47

## Data Availability

Not applicable.

## References

[1] Leardini G., Salaffi F., Caporali R., Canesi B., Rovati L., Montanelli R., Italian Group for Study of the Costs of Arthritis (2004). Direct and indirect costs of osteoarthritis of the knee. Clin. Exp. Rheumatol..

[2] Malaviya A.N., Shehab D., Bhargava S., Al-Jarallah K., Al-Awadi A., Sharma P.N., Al-Ghuriear S., Al-Shugayer A. (1998). Characteristics of osteoarthritis among Kuwaitis: A hospital-based study. Clin. Rheumatol..

[3] March L., Bachmeier C. (1998). Economics of osteoarthritis: A global perspective. Occup. Health Ind. Med..

[4] Woolf A.D., Pfleger B. (2003). Burden of major musculoskeletal conditions. Bull. World Health Organ..

[5] Knecht S., Vanwanseele B., Stüssi E. (2006). A review on the mechanical quality of articular cartilage—Implications for the diagnosis of osteoarthritis. Clin. Biomech..

[6] Lee R., Kean W.F. (2012). Obesity and knee osteoarthritis. Inflammopharmacology.

[7] Mora J.C., Przkora R., Cruz-Almeida Y. (2018). Knee osteoarthritis: Pathophysiology and current treatment modalities. J. Pain Res..

[8] Asay J.L., Erhart-Hledik J.C., Andriacchi T.P. (2018). Changes in the total knee joint moment in patients with medial compartment knee osteoarthritis over 5 years. J. Orthop. Res..

[9] Cudejko T., Van Der Esch M., Schrijvers J., Richards R., van den Noort J.C., Wrigley T., Van Der Leeden M., Roorda L.D., Lems W., Harlaar J. (2018). The immediate effect of a soft knee brace on dynamic knee instability in persons with knee osteoarthritis. Rheumatology.

[10] Holsgaard-Larsen A., Clausen B., Søndergaard J., Christensen R., Andriacchi T., Roos E. (2016). The effect on knee-joint load of instruction in analgesic use compared with neuromuscular exercise in patients with early knee osteoarthritis–A randomized, single-blind, controlled trial. Osteoarthr. Cartil..

[11] Shakoor N., Sengupta M., Foucher K., Wimmer M.A., Fogg L.F., Block J. (2010). Effects of common footwear on joint loading in osteoarthritis of the knee. Arthritis Care Res..

[12] Blagojevic M., Jinks C., Jeffery A., Jordan K. (2010). Risk factors for onset of osteoarthritis of the knee in older adults: A systematic review and meta-analysis. Osteoarthr. Cartil..

[13] Browning R.C., Kram R. (2007). Effects of Obesity on the Biomechanics of Walking at Different Speeds. Med. Sci. Sports Exerc..

[14] DeVita P., Hortobágyi T. (2003). Obesity is not associated with increased knee joint torque and power during level walking. J. Biomech..

[15] Silvernail J.F., Milner C.E., Thompson D., Zhang S., Zhao X. (2013). The influence of body mass index and velocity on knee biomechanics during walking. Gait Posture.

[16] Jeong Y., Heo S., Lee G., Park W. (2018). Pre-obesity and obesity impacts on passive joint range of motion. Ergonomics.

[17] Messier S.P. (2008). Obesity and Osteoarthritis: Disease Genesis and Nonpharmacologic Weight Management. Rheum. Dis. Clin. North Am..

[18] Runhaar J., Koes B.W., Clockaerts S., Bierma-Zeinstra S.M.A. (2011). A systematic review on changed biomechanics of lower extremities in obese individuals: A possible role in development of osteoarthritis. Obes. Rev..

[19] Haight D.J., Lerner Z.F., Board W.J., Browning R.C. (2014). A comparison of slow, uphill and fast, level walking on lower extremity biomechanics and tibiofemoral joint loading in obese and nonobese adults. J. Orthop. Res..

[20] Harding G.T., Dunbar M.J., Hubley-Kozey C.L., Stanish W.D., Wilson J.L.A. (2016). Obesity is associated with higher absolute tibiofemoral contact and muscle forces during gait with and without knee osteoarthritis. Clin. Biomech..

[21] Harding G.T., Hubley-Kozey C., Dunbar M.J., Stanish W.D., Wilson J.A. (2012). Body mass index affects knee joint mechanics during gait differently with and without moderate knee osteoarthritis. Osteoarthr. Cartil..

[22] Horsak B., Schwab C., Baca A., Greber-Platzer S., Kreissl A., Nehrer S., Keilani M., Crevenna R., Kranzl A., Wondrasch B. (2019). Effects of a lower extremity exercise program on gait biomechanics and clinical outcomes in children and adolescents with obesity: A randomized controlled trial. Gait Posture.

[23] Lerner Z.F., Board W.J., Browning R.C. (2014). Effects of obesity on lower extremity muscle function during walking at two speeds. Gait Posture.

[24] Paterson K., Sosdian L., Hinman R., Wrigley T., Kasza J., Dowsey M., Choong P., Bennell K. (2018). Effects of sex and obesity on gait biomechanics before and six months after total knee arthroplasty: A longitudinal cohort study. Gait Posture.

[25] Russell E.M., Hamill J. (2011). Lateral wedges decrease biomechanical risk factors for knee osteoarthritis in obese women. J. Biomech..

[26] Verlaan L., Boekesteijn R.J., Oomen P.W., Liu W.-Y., Peters M.J.M., Witlox M.A., Emans P.J., van Rhijn L.W., Meijer K. (2018). Biomechanical Alterations during Sit-to-Stand Transfer Are Caused by a Synergy between Knee Osteoarthritis and Obesity. Biomed. Res. Int..

[27] Yocum D., Weinhandl J.T., Fairbrother J.T., Zhang S. (2018). Wide step width reduces knee abduction moment of obese adults during stair negotiation. J. Biomech..

[28] Marouane H., Adouni M., Shirazi-Adl A. (2017). 3D active-passive response of human knee joint in gait is markedly altered when simulated as a planar 2D joint. Biomech. Model. Mechanobiol..

[29] Erdemir A. (2016). Open knee: Open source modeling & simulation to enable scientific discovery and clinical care in knee biomechanics. J. Knee Surg..

[30] Donahue T.L.H., Hull M.L., Rashid M.M., Jacobs C.R. (2002). A Finite Element Model of the Human Knee Joint for the Study of Tibio-Femoral Contact. J. Biomech. Eng..

[31] Sajjadinia S.S., Haghpanahi M., Razi M. (2019). Computational simulation of the multiphasic degeneration of the bone-cartilage unit during osteoarthritis via indentation and unconfined compression tests. Proc. Inst. Mech. Eng. H.

[32] Wilson W., Huyghe J.M., van Donkelaar C.C. (2007). Depth-dependent Compressive Equilibrium Properties of Articular Cartilage Explained by its Composition. Biomech. Model. Mechanobiol..

[33] Tissakht M., Ahmed A. (1995). Tensile stress-strain characteristics of the human meniscal material. J. Biomech..

[34] Fithian D.C., A Kelly M., Mow V.C. (1990). Material properties and structure-function relationships in the menisci. Clin. Orthop. Relat. Res..

[35] Proctor C.S., Schmidt M.B., Whipple R.R., Kelly M.A., Mow V.C. (1989). Material properties of the normal medial bovine meniscus. J. Orthop. Res..

[36] Stäubli H.U., Schatzmann L., Brunner P., Rincón L., Nolte L.-P. (1999). Mechanical Tensile Properties of the Quadriceps Tendon and Patellar Ligament in Young Adults. Am. J. Sports Med..

[37] Dhaher T.H.K., Barry M. (2010). The effect of connective tissue material uncertainties on knee joint mechanics under isolated loading conditions. J. Biomech..

[38] Limbert G., Middleton J. (2004). A transversely isotropic viscohyperelastic material: Application to the modeling of biological soft connective tissues. Int. J. Solids Struct..

[39] Adouni M., Faisal T.R., Dhaher Y.Y. (2020). Computational frame of ligament in situ strain in a full knee model. Comput. Biol. Med..

[40] Delp S.L., Anderson F.C., Arnold A.S., Loan P., Habib A., John C.T., Guendelman E., Thelen D.G. (2007). OpenSim: Open-Source Software to Create and Analyze Dynamic Simulations of Movement. IEEE Trans. Biomed. Eng..

[41] Schroeder M.J. (2014). A multi-domain synthesis of neuromechanical adaptations post anterior cruciate ligament reconstructive surgery. Biomedical Engineering.

[42] Schroeder M.J. (2010). A computational framework to evaluate the efficacy of anterior cruciate ligament reconstruction procedures. Biomedical Engineering.

[43] Russell E.M. (2011). Lateral Wedges and the Biomechanical Risk for Knee Osteoarthritis.

[44] Adouni M., Shirazi-Adl A. (2014). Partitioning of knee joint internal forces in gait is dictated by the knee adduction angle and not by the knee adduction moment. J. Biomech..

[45] Levine D., Richards J., Whittle M.W. (2012). Whittle’s Gait Analysis-E-Book.

[46] Astephen J.L. (2007). Biomechanical factors in the progression of knee osteoarthritis. School of Biomedical Engineering.

[47] MacLean K.F., Callaghan J.P., Maly M.R. (2016). Effect of obesity on knee joint biomechanics during gait in young adults. Cogent Med..

[48] Wearing S.C., Hennig E.M., Byrne N., Steele J.R., Hills A.P. (2006). Musculoskeletal disorders associated with obesity: A biomechanical perspective. Obes. Rev..

[49] Amiri P., Hubley-Kozey C., Landry S., Stanish W., Wilson J.A. (2015). Obesity is associated with prolonged activity of the quadriceps and gastrocnemii during gait. J. Electromyogr. Kinesiol..

[50] Fischer A.G., Wolf A. (2018). The effects of body weight unloading on kinetics and muscle activity of overweight males during Overground walking. Clin. Biomech..

[51] Perry J. (1992). Gait analysis: Normal and pathological function. J. Pediatric Orthop..

[52] Dufek J.S., Currie R.L., Gouws P.-L., Candela L., Gutierrez A.P., Mercer J.A., Putney L.G. (2012). Effects of overweight and obesity on walking characteristics in adolescents. Hum. Mov. Sci..

[53] Pflum M.A., Shelburne K.B., Torry M.R., Decker M.J., Pandy M.G. (2004). Model Prediction of Anterior Cruciate Ligament Force during Drop-Landings. Med. Sci. Sports Exerc..

[54] Orozco G.A., Tanska P., Mononen M., Halonen K.S., Korhonen R. (2018). The effect of constitutive representations and structural constituents of ligaments on knee joint mechanics. Sci. Rep..

[55] Shelburne K.B., Pandy M., Anderson F.C., Torry M.R. (2004). Pattern of anterior cruciate ligament force in normal walking. J. Biomech..

[56] Shelburne K.B., Torry M.R., Pandy M. (2005). Muscle, Ligament, and Joint-Contact Forces at the Knee during Walking. Med. Sci. Sports Exerc..

[57] Lacy K.W., Cracchiolo A., Yu S., Goitz H. (2016). Medial Femoral Condyle Cartilage Defect Biomechanics: Effect of Obesity, Defect Size, and Cartilage Thickness. Am. J. Sports Med..

[58] Gersing A.S., Solka M., Joseph G.B., Schwaiger B.J., Heilmeier U., Feuerriegel G., Nevitt M.C., McCulloch C.E., Link T.M. (2016). Progression of cartilage degeneration and clinical symptoms in obese and overweight individuals is dependent on the amount of weight loss: 48-month data from the Osteoarthritis Initiative. Osteoarthr. Cartil..

[59] Laberge M.A., Baum T., Virayavanich W., Nardo L., Nevitt M.C., Lynch J., McCulloch C.E., Link T.M. (2012). Obesity increases the prevalence and severity of focal knee abnormalities diagnosed using 3T MRI in middle-aged subjects—Data from the Osteoarthritis Initiative. Skelet. Radiol..

[60] Husni E. (2015). A New Look at Obesity and Osteoarthritis. https://consultqd.clevelandclinic.org/a-new-look-at-obesity-and-osteoarthritis/.

[61] Whittle M.W. (1996). Clinical gait analysis: A review. Hum. Mov. Sci..

[62] Messier S.P., Gutekunst D.J., Davis C., DeVita P. (2005). Weight loss reduces knee-joint loads in overweight and obese older adults with knee osteoarthritis. Arthritis Rheum..

[63] Mow V.C., Huiskes R. (2005). Basic Orthopaedic Biomechanics & Mechano-Biology.

[64] Henriksen M., Christensen R., Danneskiold-Samsøe B., Bliddal H. (2012). Changes in lower extremity muscle mass and muscle strength after weight loss in obese patients with knee osteoarthritis: A prospective cohort study. Arthritis Rheum..

